# Epigallocatechin-3-Gallate Suppresses BMP-6-Mediated SMAD1/5/8 Transactivation of Hepcidin Gene by Inducing SMILE in Hepatocytes

**DOI:** 10.3390/antiox10101590

**Published:** 2021-10-10

**Authors:** Yu-Ji Kim, Woo-Ram Park, Byungyoon Choi, Hueng-Sik Choi, Don-Kyu Kim

**Affiliations:** 1 Department of Integrative Food, Bioscience and Biotechnology, Chonnam National University, Gwangju 61186, Korea; call7502@naver.com (Y.-J.K.); dnfka9210@naver.com (W.-R.P.); wolfchoi1035@naver.com (B.C.); 2 School of Biological Sciences and Technology, Chonnam National University, Gwangju 61186, Korea; hsc@chonnam.ac.kr

**Keywords:** SMILE, epigallocatechin-3-gallate (EGCG), BMP-6, SMAD1/5/8, hepcidin, iron metabolism

## Abstract

Hepcidin, a major regulator of systemic iron homeostasis, is mainly induced in hepatocytes by activating bone morphogenetic protein 6 (BMP-6) signaling in response to changes in the iron status. Small heterodimer partner-interacting leucine zipper protein (SMILE), a polyphenol-inducible transcriptional co-repressor, regulates hepatic gluconeogenesis and lipogenesis. Here, we examine the epigallocatechin-3-gallate (EGCG) effect on BMP-6-mediated SMAD1/5/8 transactivation of the hepcidin gene. EGCG treatment significantly decreased BMP-6-induced hepcidin gene expression and secretion in hepatocytes, which, in turn, abated ferroportin degradation. SMILE overexpression significantly decreased BMP receptor-induced hepcidin promoter activity. SMILE overexpression also significantly suppressed BMP-6-mediated induction of hepcidin mRNA and its secretion in HepG2 and AML12 cells. EGCG treatment inhibited BMP-6-mediated hepcidin gene expression and secretion, which were significantly reversed by SMILE knockdown in hepatocytes. Interestingly, SMILE physically interacted with SMAD1 in the nucleus and significantly blocked DNA binding of the SMAD complex to the BMP-response element on the hepcidin gene promoter. Taken together, these findings suggest that SMILE is a novel transcriptional repressor of BMP-6-mediated hepcidin gene expression, thus contributing to the control of iron homeostasis.

## 1. Introduction

Hepcidin, a hepatic circulating peptide, acts as a critical regulator of systemic iron homeostasis, which is the balance between the iron uptake into enterocytes, stored iron release from hepatocytes and iron recycling by macrophages [1]. Hepcidin can bind to ferroportin (FPN, a mammalian iron exporter located mainly on the membrane of enterocytes, hepatocytes and macrophages) and induce its endocytosis and lysosomal degradation, thereby precluding iron efflux into plasma [2]. Hypoxia, erythropoiesis and various endocrine stimuli can repress hepcidin expression, while infection and inflammation can stimulate hepcidin expression [3,4,5]. Bone morphogenetic protein-6 (BMP-6) can also induce hepcidin gene expression by activating small mothers against decapentaplegic (SMADs) homolog 1, 5 and 8 proteins [6,7,8,9,10]. The BMP-6–hemojuvelin (HJV, a BMP co-receptor) complex can bind to heterodimeric BMP receptors (BMPRs) composed of type I (BMPRI; ALK1, ALK2, ALK3 and ALK6) and type II (BMPRII, ACTRIIA and ACTRIIB) serine threonine kinase receptors. Activated BMPR I can then phosphorylate regulatory SMADs (R-SMADs; SMAD1, SMAD5 and SMAD8) [7,11]. Phosphorylated SMAD1/5/8 proteins then form a heteromeric complex with co-mediator SMAD (co-SMAD; SMAD4). Then, translocation of the complex into the nucleus can activate hepcidin transcription [12,13].

Small heterodimer partner-interacting leucine zipper protein (SMILE), also known as CREBZF, is a member of the cAMP response element-binding (CREB) and activating transcription factor (ATF) subgroup of the basic region-leucine zipper (bZIP) family. It could form a homodimer incapable of DNA-binding [14]. It is known that SMILE can act as a co-regulator of several nuclear receptors (NRs), including glucocorticoid receptor, hepatocyte nuclear factor 4α (HNF4α), constitutive androstane receptor and estrogen-related receptor γ (ERRγ) by binding to the ligand-binding domain containing activation function-2 [15,16]. In the liver, SMILE can repress the expression of the CREB/CRTC2-mediated gluconeogenic gene and LXRα-mediated lipogenic gene, leading to a decrease in blood glucose and fatty liver, respectively [17,18]. Thus, SMILE is considered as an inducible transcriptional repressor of endocrine and metabolic signals, such as bile acid and insulin [18,19]. Recently, SMILE can inhibit STAT3 activation by interleukin-6 (IL-6), a proinflammatory cytokine, in hepatocytes [20]. However, whether SMILE is involved in the activation of SMADs by BMP-6, a member of the TGF-β superfamily of cytokines, leading to altered iron homeostasis remains largely unknown.

Epigallocatechin-3-gallate (EGCG), a major bioactive polyphenolic component of green tea, is known to have many beneficial biological effects, such as anti-oxidative, anti-proliferative, anti-microbial and anti-inflammatory actions, depending on numerous conditions, including dose and cell type [21,22]. EGCG can modulate several intracellular signaling cascades, such as an extracellular-regulated protein kinases and mitogen-activated protein kinases signaling, thereby regulating expression of target genes involved in enzyme activity, apoptosis, cell proliferation and metabolism [22]. Moreover, EGCG decreased sterol regulatory-element binding protein-2 expression by activating sirtuin1-forkhead box protein O1 (FOXO1) signaling, thereby inhibiting hepatic cholesterol synthesis [23]. In addition, EGCG can ameliorate glucose- and palmitate acid-induced inflammation, oxidative stress and lipid accumulation by activating the glucose transporter-2 signaling pathway, resulting in enhanced insulin sensitivity [24]. In hepatocytes, EGCG can also repress the expression of the HNF4α gene by activating ERK1/2 signaling, leading to inhibition of hepatitis B virus replication [25]. Recently, EGCG can inhibit IL-6-STAT3 signaling in hepatocytes [20]. However, the effect of EGCG on BMP-6-dependent hepcidin expression in hepatocytes remains unknown. Therefore, in this study, we examine the EGCG effect on BMP-6-mediated SMAD1/5/8 transactivation of hepcidin gene expression.

## 2. Materials and Methods

### 2.1. Chemicals

Recombinant mouse bone morphogenetic protein 6 (BMP-6, >95% purity; R&D Systems, Inc., Minneapolis, MN, USA) was dissolved in 4 mM HCl containing 0.1% bovine serum albumin. This solvent was used as a vehicle in this study unless specifically mentioned otherwise. Epigallocatechin gallate (EGCG, ≥98% purity; Tocris Bioscience, Bristol, UK) was dissolved in deionized water.

### 2.2. Plasmid DNA and Recombinant Adenovirus Construction

Promoters of mouse (−982/+84 bp, NM_032541) and human (−2762 bp, NM_021175) hepcidin genes were described previously [26,27]. pcDNA3-FLAG-SMILE (NM_001039618.4), pcDNA3-HA-SMILE, pEGFP-SMILE, pCMV-SPORT6-HFE2 (BC022603), pcDNA3-Myc-SMAD1 (NM_005900.3), pcDNA3-Myc-SMAD5 (NM_008541.3), pcDNA3-Myc-SMAD8 (XM_039103275.1) and pcDNA3-HA-SMAD4 (NM_005359) were obtained as indicated previously [28,29]. To obtain HA tagged-SMAD1, -SMAD5 and -SMAD8 constructs, each SMAD gene was amplified by polymerase chain reaction (PCR) from pcDNA3-Myc-SMAD1, pcDNA3-Myc-SMAD5 and pcDNA3-Myc-SMAD8 and then subcloned into a pcDNA3-HA vector using *EcoR* V and *Xho* I restriction sites, respectively. pcDNA3-ALK3 constitutively active form (ALK3-CA, NM_004329) and pCMV5B-FLAG-SMAD1 were kindly provided by Dr. Carl-Henrik Heldin (Uppsala University) and Dr. Jeff Wrana (Lunenfeld-Tanenbaum Research Institute), respectively. Adenovirus-encoding green fluorescent protein (GFP) (Ad-GFP), SMILE (Ad-SMILE), unspecific short hairpin RNA (shRNA) for control (Ad-US) and sh-SMILE (Ad-shSMILE) were obtained as described previously and amplified using AD293 cells (ATCC, Manassas, VA, USA) [28,30].

### 2.3. Cell Culture and Transient Transfection

Human hepatoma cell lines (HepG2 and Huh7 cells) and a mouse immortalized hepatocyte cell line (AML12) were obtained from the ATCC and cultured as previously described [20]. SuperFect (QIAGEN, Hilden, Germany) or polyethylenimine (Polysciences, Inc., Warrington, PA, USA) were used for transient transfections according to each manufacturer’s recommendations. Small interfering RNAs (si-Con and si-SMILE) (QIAGEN) were transfected into HepG2 cells using Lipofectamine RNAi MAX (Thermo Fisher Scientific, Waltham, MA, USA). The luciferase assay was carried out as described previously [31]. In brief, HepG2 cells were transfected with indicated reporter plasmids together with vectors expressing SMILE, ALK3-CA, or HFE2 followed by treatment with BMP-6 or EGCG for 12 h.

### 2.4. Cell Viability Assay

Cell viability was measured using an MTT-based assay, as previously described [20]. Briefly, HepG2, Huh7 and AML12 cells were treated with EGCG for 12 h and treated with an indicated concentration of EGCG from different time periods after pre-treatment with BMP-6 for 12 h. To evaluate long-term cell viability, HepG2 and AML12 cells were treated with EGCG up to 96 h. The viability of treated cells was calculated by comparing the absorbance of treated cells to the absorbance of control cells (set to have 100% viability).

### 2.5. Quantitative PCR (Q-PCR) Analysis

HepG2 and AML12 cells infected with Ad-GFP or Ad-SMILE were exposed to BMP-6 or EGCG for 12 h. Adenoviral infections (multiplicity of infection, MOI) and treatment with BMP-6 or EGCG were performed as described in figure legends. Total RNAs were reverse-transcribed into complementary DNAs (cDNAs) and analyzed using quantitative PCR (Q-PCR) [20]. All data were normalized to L32 or β-actin. Gene expression levels were analyzed using the comparative cycle threshold (2^−ΔΔCt^) method.

### 2.6. Western Blot (WB) Analysis

The WB analysis was carried out as previously described [20,32]. Briefly, proteins from whole-cell lysates and fractions were separated by 10% SDS-polyacrylamide gel electrophoresis and transferred to Amersham nitrocellulose membranes (GE Healthcare, IL, USA). These membranes were incubated with the following primary antibodies: anti-DYKDDDDK (FLAG, 1:2000 dilution; Invitrogen, CA, USA), anti-HA (1:3000 dilution; Santa Cruz Biotechnology, Dallas, TX, USA), anti-Lamin A/C (1:3000 dilution; Santa Cruz Biotechnology), anti-α-tubulin (1:2000 dilution; Santa Cruz Biotechnology), anti-HA (1:500 or 1:3000 dilution; Cell signaling Technology), anti-Actin (1:3000 dilution; Santa Cruz Biotechnology), anti-Ferroportin (1:3000 dilution; Novus Biologicals, Centennial, CO, USA) and anti-SMILE (Zhangfei, 1:1000 or 1:3000 dilution; Abcam, Cambridge, UK). Primary antibodies were then probed with horseradish peroxidase-conjugated secondary antibodies (1:3000 dilution; Bethyl Laboratories, Montgomery, TX, USA). Images were visualized using ECL reagents (GE Healthcare).

### 2.7. Immunocytochemical Analysis

HepG2 cells were fixed with ice-cold acetone at room temperature for 5 min, washed three times with ice-cold phosphate buffered saline (PBS) supplemented with 0.1% Tween 20 (PBST) and blocked with PBST supplemented with 1% bovine serum albumin and 22.52 mg/mL glycine at room temperature for 30 min. Immunofluorescence staining was described previously [20].

### 2.8. Co-Immunoprecipitation (co-IP) Analysis

The Co-IP analysis was performed using a FLAG IP kit (Sigma). Briefly, HepG2 cells were transfected with FLAG-SMILE, HA-SMAD1, HA-SMAD5, HA-SMAD8 and HA-SMAD4 for 48 h. Whole-cell lysates (200 μL) were immunoprecipitated with anti-FLAG M2 affinity gel and eluted with 3× FLAG peptide. The WB analysis was performed using anti-HA (Cell signaling Technology) and anti-FLAG antibodies (Invitrogen).

### 2.9. Chromatin Immunoprecipitation (ChIP) Assay

The ChIP assay was carried out according to the manufacturer’s instructions (Cell Signaling Technology). Soluble chromatins were isolated from HepG2 cells transfected with mouse hepcidin gene promoter (mHepcidin-luc), HA-SMILE and FLAG-SMAD1 and treated with BMP-6 for 12 h. After recovering DNA, Q-PCR was carried out using BMP-RE primers. Primer sequences were described previously [29].

### 2.10. Measurement of Hepcidin Levels

Hepcidin levels in cell culture media were measured using a human (R&D Systems) and mouse (Elabscience, TX, USA) hepcidin enzyme-linked immunosorbent assay (ELISA) kit according to each manufacturer’s instructions, respectively.

### 2.11. Statistics

All data are represented as means ± standard deviation (SD) and were analyzed by GraphPad Prism (GraphPad Software, CA, USA) two-tailed Student’s *t*-test. Statistical differences were considered at the 0.05 levels of probability (*p* < 0.05).

## 3. Results

### 3.1. EGCG Suppresses BMP-6 Receptor Signaling in Hepatocytes

**Figure 1:**
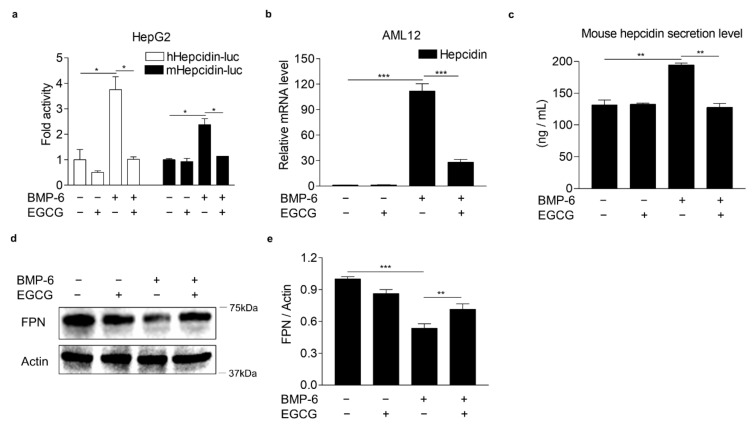
EGCG suppresses BMP-6-induced hepcidin expression and secretion. (**a**) EGCG effect on BMP-6-mediated hepcidin gene promoter activity. HepG2 cells transfected with hepcidin gene promoters were exposed to EGCG (+, 100 μM) and BMP-6 (+, 20 nM) for 12 h. (**b**) EGCG effect on BMP-6-induced hepcidin gene expression. (**c**) Inhibitory effect of EGCG on secreted hepcidin levels induced by BMP-6. Secreted hepcidin levels in cell culture media were measured using ELISA. (**d**) Effect of EGCG on ferroportin (FPN) expression reduced by BMP-6. All gels for WB analysis were run under the same experimental conditions. (**e**) Graphical analysis of FPN expression. AML12 cells were co-treated with EGCG (+, 100 μM) and BMP-6 (+, 20 nM) for 12 h. Data are representative of at least three independent experiments. Data are expressed as means ± SD. * *p* < 0.05, ** *p* < 0.01, *** *p* < 0.001 by two-tailed Student’s *t*-test.

To investigate whether EGCG could affect BMP-6 receptor signaling in hepatocytes, we first examined the cytotoxic effects of EGCG on human (HepG2 and Huh7 cells) and mouse (AML12 cells) hepatocytes using the MTT assay. As a result, EGCG was not cytotoxic to both hepatocytes for 24 h at a concentration of 100 μM used throughout this study (Figure S1). In addition, 100 μM of EGCG or 20 nM of BMP-6 plus 100 μM of EGCG treatment for 24 h did not affect the viability of hepatocytes (Figure S2). Moreover, recombinant mouse BMP-6 significantly increased SMAD1/5/8 phosphorylation in Hu7 and HepG2 cells, as well as AML12 cells (Figure S3), indicating that mouse BMP-6 has cross reactivity to human BMP-6 receptors. Next, to examine the effect of EGCG on BMP-6-mediated hepcidin expression, HepG2 cells were transfected with the hepcidin gene promoter and exposed to BMP-6 and EGCG. As a result, BMP-6 treatment increased the activity of both human and mouse hepcidin gene promoters, which were significantly decreased by EGCG treatment (Figure 1a). In addition, hepcidin mRNA and secretion levels were measured in AML12 cells co-treated with BMP-6 and EGCG. As expected, BMP-6 treatment significantly induced hepcidin mRNA expression (Figure 1b). However, such hepcidin induction was strongly inhibited by EGCG treatment. Consistent with these findings, BMP-6-induced hepcidin secretion levels were decreased to control levels following EGCG treatment (Figure 1c). Hepcidin has been reported to be able to bind to FPN, an iron export protein, leading to its internalization and lysosomal degradation [33]. Thus, we tested if EGCG could affect FPN expression in AML12 cells co-treated with BMP-6 and EGCG. As expected, the reduction of FPN by BMP-6 was significantly reversed by EGCG (Figure 1d,e). These results suggest that EGCG could inhibit BMP-6 receptor signaling in hepatocytes.

### 3.2. EGCG-Inducible SMILE Represses BMP-6 Receptor Signaling

**Figure 2:**
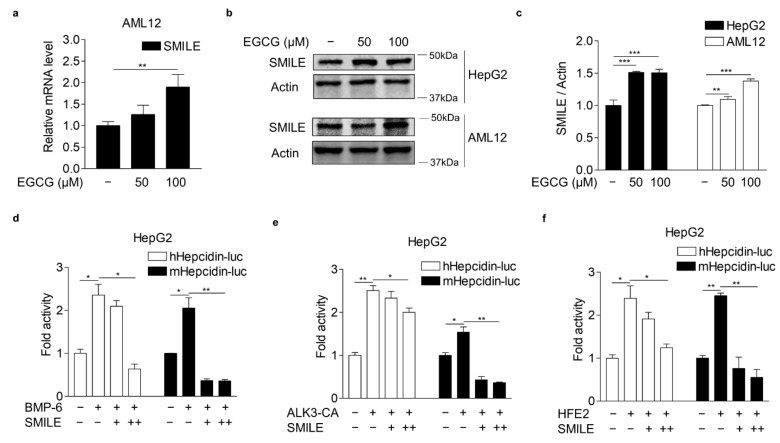
EGCG-inducible SMILE inhibits hepcidin promoter activity mediated by BMP-6 signaling. (**a**) Q-PCR analysis showing EGCG effect on SMILE mRNA levels in AML12 cells treated with different concentrations of EGCG for 12 h. (**b**) WB analysis showing EGCG effect on SMILE protein expression in AML12 and HepG2 cells exposed to different concentrations of EGCG for 12 h. (**c**) Graphical analysis of SMILE expression in HepG2 and AML12 cells. (**d**) SMILE effect on BMP-6-induced hepcidin gene promoter activity. HepG2 cells co-transfected with hepcidin gene promoters and SMILE were stimulated with BMP-6 (+, 20 nM) for 12 h. (**e**) SMILE effect on ALK3-induced hepcidin gene promoter activity. (**f**) SMILE effect on HFE2-mediated hepcidin gene promoter activity. Data are representative of at least three independent experiments. Data are expressed as means ± SD. * *p* < 0.05, ** *p* < 0.01, *** *p* < 0.001 by two-tailed Student’s *t*-test.

SMILE has been reported to be critical for EGCG-mediated inhibition of IL-6 receptor signaling in hepatocytes [20]. Indeed, EGCG significantly increased SMILE mRNA and protein levels as demonstrated by Q-PCR and WB analysis, respectively, in AML12 and HepG2 cells (Figure 2a–c). These results raised the possibility that SMILE might play a key role in the inhibitory effect of EGCG on BMP-6 receptor signaling in hepatocytes. Therefore, we next examined whether SMILE could inhibit BMP-6 receptor signaling in HepG2 cells transiently transfected with the hepcidin gene promoter and SMILE and treated with BMP-6. As expected, SMILE significantly decreased promoter activity of human and mouse hepcidin genes increased by BMP-6 stimulation (Figure 2d). It has been reported that ALK3, a BMP receptor type I, is also important for the induction of hepcidin gene expression in hepatocytes [34,35]. We tested if SMILE could inhibit ALK3-induced hepcidin promoter activity in HepG2 cells. Induction of hepcidin promoter activities by co-transfection with a constitutively active form ALK3 (ALK3-CA) was significantly reduced by co-transfection with SMILE (Figure 2e). HFE2 (encoding hemojuvelin, also known as HJV) can act as a BMP coreceptor to enhance hepcidin expression by activating BMP signaling [7,8]. Indeed, HFE2 overexpression significantly increased human and mouse hepcidin promoter activities. However, these activities were reduced to control levels following co-transfection with SMILE (Figure 2f). These results indicate that EGCG-inducible SMILE can inhibit BMP signaling, which resulted in hepcidin induction.

### 3.3. SMILE Inhibits BMP-6-Induced Hepcidin Expression

**Figure 3:**
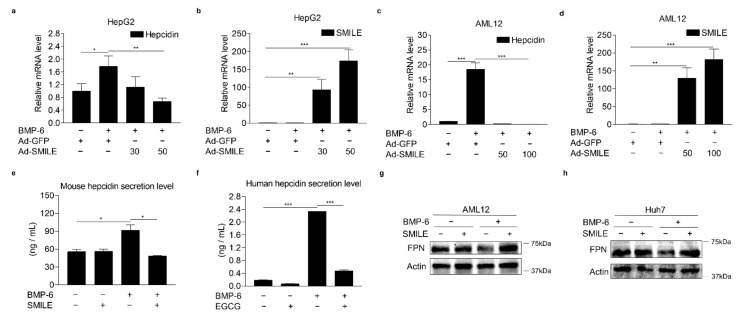
SMILE represses BMP-6-mediated hepcidin expression and secretion. (**a**,**b**) Effect of SMILE on BMP-6-induced hepcidin mRNA expression in HepG2 cells. Hepcidin mRNA levels (**a**) and SMILE mRNA levels (**b**) were measured by Q-PCR analysis. Adenoviral overexpression of SMILE (30 and 50 MOI) or GFP (+, 30 MOI) was performed in HepG2 cells for 36 h and the cells exposed to BMP-6 (+, 20 nM) for 12 h. (**c**,**d**) Effect of SMILE on BMP-6-induced hepcidin mRNA expression in AML2 cells. Hepcidin mRNA levels (**c**) and SMILE mRNA levels (**d**) were measured by Q-PCR analysis. Adenoviral overexpression of SMILE (50 and 100 MOI) or GFP (+, 50 MOI) was performed in AML12 cells for 36 h and the cells exposed to BMP-6 (+, 20 nM) for 12 h. (**e**,**f**) Effect of SMILE on secreted hepcidin levels induced by BMP-6. SMILE was transfected into AML12 (**e**) and Huh7 (**f**) cells. After 2 h serum starvation, these cells were stimulated with BMP-6 (+, 20 nM) for 12 h. Secreted hepcidin levels in cell culture media were measured using ELISA. (**g**,**h**) Effect of SMILE on BMP-6-mediated FPN expression. SMILE was transfected into AML12 and Huh7 cells and then these cells were stimulated with BMP-6 (+, 20 nM) for 12 h. Data are representative of at least three independent experiments. Data are expressed as means ± SD. * *p* < 0.05, ** *p* < 0.01, *** *p* < 0.001 by two-tailed Student’s *t*-test.

To further examine whether SMILE could inhibit BMP-6-mediated hepcidin gene transcription, AML12 and HepG2 cells were infected with Ad-GFP or Ad-SMILE and then treated with BMP-6. Consistent with results of the transient transfection analysis, BMP-6 significantly increased hepcidin mRNA levels in HepG2 cells. However, such increases were strongly inhibited by SMILE overexpression (Figure 3a,b). In addition, BMP-6-mediated hepcidin expression was reduced to control levels by overexpressing SMILE in AML12 cells (Figure 3c,d). Indeed, SMILE overexpression markedly suppressed BMP-6-induced hepcidin secretion from both human and mouse hepatocyte cells (Figure 3e,f). These results were further supported by results showing that SMILE normalized the reduction of FPN protein levels by BMP-6 in mouse and human hepatocytes (Figure 3g,h). Taken together, these results imply that SMILE can act as a transcriptional repressor of BMP-6-induced hepcidin expression.

### 3.4. SMILE Is Required for the Inhibitory Effect of EGCG on BMP-6-Mediated Hepcidin Expression

**Figure 4:**
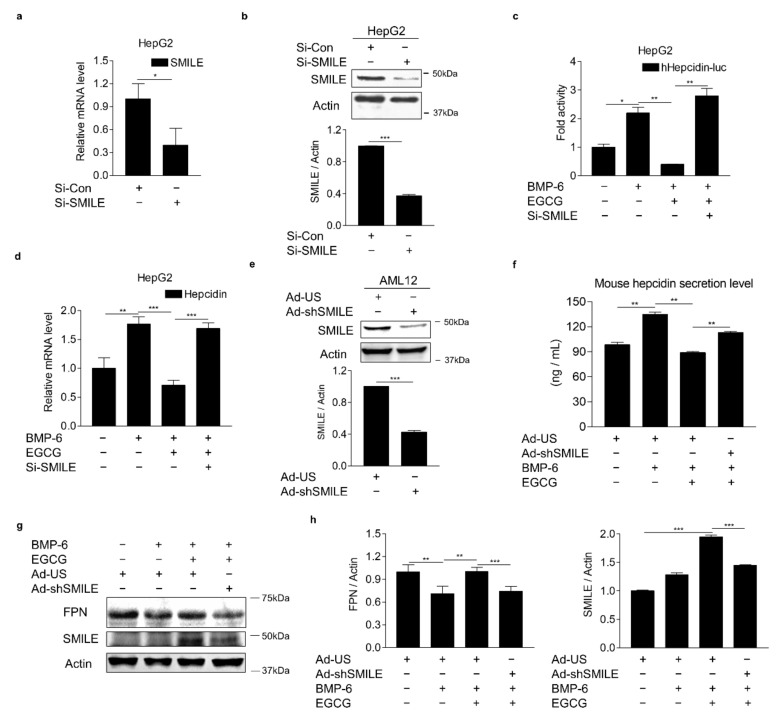
SMILE knockdown suppresses effects of EGCG on BMP-6-mediated hepcidin expression and secretion. (**a**,**b**) Efficiency of SMILE knockdown at mRNA and protein levels in HepG2 cells. (**c**) SMILE knockdown blocks EGCG-mediated reduction of hepcidin gene promoter activity. After co-transfection of human hepcidin gene promoter and si-SMILE, HepG2 cells were co-treated with EGCG (+, 100 μM) and BMP-6 (+, 20 nM) for 12 h. (**d**) SMILE knockdown blocks EGCG-mediated reduction of hepcidin gene expression. After transient transfection of si-SMILE, HepG2 cells were co-treated with EGCG (+, 100 μM) and BMP-6 (+, 20 nM) for 12 h. (**e**) Efficiency of SMILE knockdown at protein levels. AML12 cells infected with Ad-shSMILE or Ad-US for 24 h. (**f**) SMILE knockdown effects on secreted hepcidin levels reduced by EGCG. (**g**) SMILE knockdown effects on FPN expression mediated by EGCG. (**h**) Graphical analysis of FPN (**left**) and SMILE (**right**) expression. AML12 cells were exposed to EGCG (+, 100 μM) and BMP-6 (+, 20 nM) for 12 h after infection with Ad-US or Ad-shSMILE (+, 50 MOI, respectively) for 24 h. Data are representative of at least three independent experiments. Data are expressed as means ± SD. * *p* < 0.05, ** *p* < 0.01, *** *p* < 0.001 by two-tailed Student’s *t*-test.

To verify the involvement of SMILE in the effect of EGCG on BMP-6-mediated hepcidin expression, loss-of-function studies using SMILE-targeting small interfering RNAs (si-SMILE) or adenoviral overexpression of short-hairpin SMILE (Ad-shSMILE) were performed in hepatocytes. We first examined the knockdown efficiency of SMILE in HepG2 cells transfected with siCon or siSMILE. Results showed that both mRNA and protein levels of SMILE were significantly decreased by siSMILE compared to those in the control (Figure 4a,b). Consequently, SMILE knockdown entirely abolished the inhibitory effect of EGCG on BMP-6-induced hepcidin gene promoter activity (Figure 4c). Moreover, EGCG significantly suppressed BMP-6-induced hepcidin mRNA levels. However, such suppression was almost completely abrogated by SMILE knockdown (Figure 4d). To further examine whether SMILE knockdown could reverse the inhibitory effect of EGCG on BMP-6-mediated hepcidin secretion, AML12 cells were infected with either Ad-US or Ad-shSMILE and then treated with BMP-6 and EGCG. As expected, the endogenous SMILE blockade by Ad-shSMILE significantly attenuated the inhibitory effect of EGCG on BMP-6-induced hepcidin secretion (Figure 4e,f). As a result of hepcidin induction by BMP-6, FPN protein levels were decreased. However, they returned to normal levels after treatment with EGCG (Figure 4g,h). On the other hand, the EGCG effect on FPN was almost entirely blocked by SMILE knockdown. Overall, these results imply that SMILE is required for the EGCG effect on BMP-6-mediated hepcidin gene expression in hepatocytes.

### 3.5. SMILE Interacts with SMAD1 and Inhibits Its DNA-Binding to Hepcidin Gene Promoter

**Figure 5:**
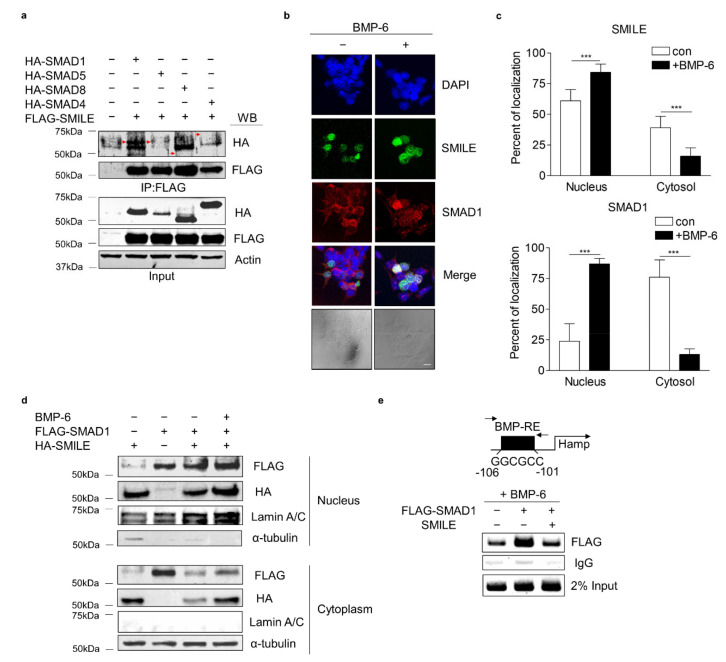
SMILE physically interacts with SMAD1 and diminishes SMAD1 binding activity to hepcidin gene promoter. (**a**) Co-IP analysis showing interactions between SMADs and SMILE. HA-SMAD1, HA-SMAD5, HA-SMAD8 or HA-SMAD4 and FLAG-SMILE were transfected into HepG2 cells. Red arrows indicate HA-SMADs. (**b**) Immunocytochemical analysis showing subcellular co-localization of SMILE and SMAD1. Scale bar = 10 μm. (**c**) Relative fluorescence of SMILE (top) and SMAD1 (bottom) in the nucleus and cytoplasm. HepG2 cells were exposed to BMP-6 (+, 20 nM) for 12 h after transient transfection of FLAG-SMAD1 and GFP-SMILE. Graphical quantifications were performed using at least sixteen transfected cells. (**d**) Subcellular localization of SMAD1 and SMILE. After transient transfection of FLAG-SMAD1 and HA-SMILE, HepG2 cells were exposed to BMP-6 (+, 20 nM) for 12 h. (**e**) SMILE inhibits SMAD1 binding to hepcidin gene promoter. After co-transfection of mouse hepcidin gene promoter, FLAG-SMAD1 and HA-SMILE, HepG2 cells were exposed to BMP-6 (+, 20 nM) for 12 h. Soluble chromatins were immunoprecipitated using anti-IgG or anti-FLAG antibodies. ChIP samples were amplified by PCR. BMP-RE, BMP-response element. Data are expressed as means ± SD. *** *p* < 0.001 by two-tailed Student’s *t*-test.

To explore the molecular mechanism underlying the inhibitory effect of SMILE on BMP-6 receptor signaling in hepatocytes, co-immunoprecipitation (co-IP) and WB analyses were performed in HepG2 cells co-transfected with vectors encoding SMILE and SMADs (SMAD1, SMAD5, SMAD8 and SMAD4). Interestingly, SMILE interacted with SMAD1, but not with the others (Figure 5a). Based on the interaction between SMILE and SMAD1, the subcellular localizations of SMILE and SMAD1 were investigated. Immunocytochemistry analyses of HepG2 cells were performed in the presence or absence of BMP-6. In the absence of BMP-6, SMAD1 was localized mainly in cytoplasm and SMILE was localized in the nucleus (Figure 5b,c). However, in the presence of BMP-6, SMAD1 and SMILE co-localized mainly in the nucleus (Figure 5c). A subcellular fractionation analysis was carried out using HepG2 cells transfected with vectors encoding SMILE and SMAD1 in the presence or absence of BMP-6. BMP-6 markedly enhanced the interaction between SMILE and SMAD3 in the nucleus (Figure 5d). Finally, the chromatin immunoprecipitation (ChIP) assay revealed that SMAD1 could strongly bind to a BMP-response element (BMP-RE) on the hepcidin gene promoter in the presence of BMP-6. However, this binding was completely blocked by overexpression of SMILE (Figure 5e). These findings demonstrate that SMILE can inhibit BMP-6-mediated hepcidin transcription by interfering with DNA-binding of SMAD1 to the hepcidin gene promoter.

## 4. Discussion

**Figure 6:**
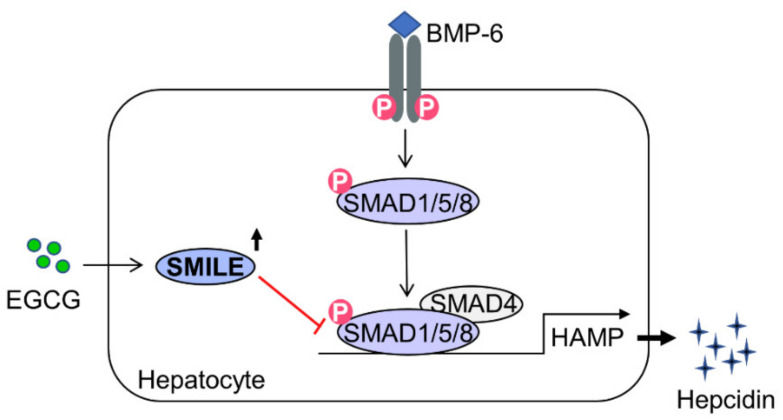
A schematic diagram showing mechanism involved in the inhibition effect of EGCG on BMP-6-mediated hepcidin expression by inducing SMILE expression. In hepatocytes, EGCG-inducible SMILE inhibits BMP-6-mediated SMAD1 transactivation to hepcidin gene promoter, resulting in decrease in hepcidin production and secretion.

Iron-deficiency anemia is the most frequent type of anemia worldwide. It is mainly treated with oral iron supplementation (such as ferrous sulfate) or intravenous (IV) iron injection (such as ferric gluconate) [36,37]. Unfortunately, therapy with oral iron supplementation or IV iron injection is ineffective for hepcidinopathies known to be iron metabolism disorders triggered by dysregulating hepcidin, such as iron-refractory iron deficiency anemia (IRIDA) and anemia of inflammation [37,38]. IRIDA is one type of iron-deficiency anemia due to mutation of the transmembrane serine protease 6, which cleaves HJV and, in turn, inhibits BMP-6-induced hepcidin expression [39]. Thus far, hepcidin, a key regulator of iron metabolism, has been considered as not only an indicator, but also a promising therapeutic target of iron metabolism disorders. Indeed, antagonists including PRS-080, NOX-H94 and LY2928057 (a humanized monoclonal FPN antibody) can improve serum iron levels and transferrin saturation in clinical tests with healthy and chronic kidney disease patients [38]. Besides, by exploring molecular mechanisms, several drugs that target hepcidin expression are under steady research and development. For example, erythroferrone, testosterone, anticoagulant heparin and synthetic compounds, such as LY3113593 (a monoclonal antibody targeting BMP-6), H5F9-AM8 (an HJV-neutralizing antibody) and LDN-193189 (a selective antagonist of BMP receptor isotypes ALK2 and ALK3), blocked the BMP-6–HJV–SMADs signaling pathway, consequently leading to inhibition of hepcidin expression and restoration of serum iron levels [38]. Interestingly, myricetin, a natural dietary flavonoid, can suppress BMP-6- and IL-6-induced hepcidin expression, thus alleviating lipopolysaccharide-induced hypoferremia [40]. In this study, EGCG could significantly suppress BMP-6-induced hepcidin expression and consequently normalize hepcidin-mediated FPN expression through induction of SMILE expression in hepatocytes (Figure 6). Interestingly, SMILE is also considered a polyphenol-inducible transcriptional co-repressor, since SMILE expression is significantly upregulated by polyphenolic compounds such as curcumin and resveratrol in hepatocytes [16,30]. This notion is further supported by findings showing that SMILE is significantly induced by EGCG and that it can inhibit IL-6-STAT3 signaling in hepatocytes [20]. These findings suggest that SMILE is important for transcriptional repression of hepcidin gene expression triggered by both BMP-6-SMAD1/5/8 and IL-6-STAT3 signaling in hepatocytes.

In this study, we reveal a previously unrecognized effect of EGCG in BMP-6-mediated hepcidin expression using in vitro cell culture models. EGCG (100 μM) significantly inhibited hepcidin expression induced by BMP-6 in HepG2 and AML12 cells. These results suggest a biomedical potential of EGCG to control abnormal regulation of iron homeostasis in vivo. Indeed, in iron overload with β-thalassemia, EGCG (50 mg/kg) abated oxidative hepatic tissue damage and β-cell impairment by boosting the iron chelation therapy of deferiprone leading to a reduction in tissue iron levels [41]. Previously, treatment of EGCG (100 mg/kg) decreased hepcidin secretion and hypoferremia induced by lipopolysaccharide in the liver of mice [20]. These findings suggest that EGCG may have a biomedical potential to control iron-related cellular and physiological processes at relatively high concentrations. However, it is reported that pharmacokinetic studies showed that physiological serum concentration of EGCG is in the nanomolar ranges in humans [42,43,44]. Accordingly, high micromolar concentrations are unlikely to be in the blood of individuals that drink green tea or ingest green tea extract. However, we cannot exclude the possibility that EGCG may be accumulated in tissues such as the liver to generate intracellular concentrations that are much higher than those observed in clinical human serum samples. In addition, treatment with high doses of EGCG at a daily dosage of 856.8 mg for 12 weeks showed anti-obesity effects without any side effect in human obesity [45]. It is also likely that EGCG may be metabolized and produce more potent and effective bioactive forms. Moreover, EGCG may elicit a positive synergistic effect when treated with other catechins [46]. Therefore, the in vivo effect of high concentration of EGCG on disorders of iron metabolism needs to be further investigated.

The BMP-6 and IL-6 signaling pathways are closely interconnected in the regulation of hepcidin expression. It has been reported that mutations of BMP-RE1 (-84/-79) located close to the STAT3-binding site (-72/-64) in the hepcidin gene promoter can abrogate IL-6-induced hepcidin gene promoter activity [47]. Furthermore, treatment with LDN-193189, noggin and ALK3-Fc (BMP antagonists) can inhibit IL-6-induced hepcidin expression, leading to improved turpentine-induced anemia [48]. Previously, it is reported that GSK5182, an ERRγ inverse agonist, can inhibit IL-6-mediated hepcidin expression caused by infection of *Salmonella typhimurium* in hepatocytes, indicating that ERRγ can act as a transcriptional mediator in IL-6-mediated hepcidin induction [27]. Recently, it has been reported that hepatocyte-specific ERRγ knock-out or treatment with GSK5182 can repress IL-6-induced BMP-6 expression in hepatocytes, indicating that IL-6-induced ERRγ can directly enhance hepatic BMP-6 production [49]. These findings imply that ERRγ plays a key role in the crosstalk between BMP-6 and IL-6 signaling on hepcidin production. Interestingly, SMILE is required for GSK5182-mediated inhibition of ERRγ activity by recruiting SIRT1 [16]. In addition, SMILE induced by curcumin in hepatocytes can repress transactivation of CREBH on hepcidin expression [30]. These findings suggest that SMILE is critical for the regulation of hepcidin expression and iron homeostasis.

Excessive hepcidin not only causes anemia by restricting systemic iron availability, but also induces intracellular ROS by sequestrating iron into cells, supporting that iron metabolism is implicated in redox homeostasis [1,50]. For example, activated NFE2-related factor 2, by sensing systemic iron accumulation or by treatment with EGCG, can induce BMP-6 and hepcidin expression by binding to the antioxidant response element on the gene promoter, subsequently decreasing serum iron levels and iron toxicity [51,52]. Interestingly, it is reported that hepatocellular iron accumulation inhibited hepcidin expression by suppressing BMP/SMAD and IL-6/STAT3 signaling [53]. Our previous findings have shown that EGCG at a high concentration can diminish IL-6-induced hepcidin expression and secretion through the induction of FOXO1-dependent SMILE expression, which, in turn, can increase serum iron levels [20]. On the other hand, it has also been reported that a high dose of green tea extract containing high concentrations of active EGCG can decrease hepatic iron accumulation by reducing hepcidin expression in the serum, thus ameliorating hepatic fibrogenesis and hepatotoxicity by iron overload [54]. Supported by the above-mentioned report, through a loss-of-SMILE-function study using si-SMILE or Ad-shSMILE, we demonstrated that EGCG significantly down-regulated BMP-6-induced hepcidin production and secretion through the induction of SMILE expression and subsequently recovered FPN expression. These results suggest that EGCG can regulate hepcidin gene expression by redox-dependent functional switching, consequently leading to maintenance of iron homeostasis.

Interestingly, EGCG induces FOXO1 activity in hepatocytes [23], indicating that FOXO1 is a downstream mediator of EGCG. Previously, EGCG significantly increased gene transcription of hepatic SMILE through the induction of FOXO1 expression, indicating that the FOXO1 is critical in EGCG-induced SMILE expression [20]. In addition, EGCG activates ROS-mediated liver kinase B1 (LKB1) resulting in AMP-dependent kinase (AMPK) activation in hepatocytes [55]. These results are further supported by the report showing that a polyphenol curcumin activates the LKB1–AMPK axis resulting in SMILE induction in hepatocytes [30]. Moreover, it is reported that FOXO1 activity is upregulated by LKB1-induced AMPK activation [56,57,58]. Taken together, these findings suggest that EGCG upregulates FOXO1 through the activation of LKB1–AMPK signaling.

## 5. Conclusions

In conclusion, we demonstrate a previously unrecognized role of SMILE in BMP-6-mediated hepcidin expression in hepatocytes with detailed molecular mechanisms involved in this process. EGCG-inducible SMILE suppressed BMP-6-induced hepcidin expression and secretion by inhibiting SMAD1-binding to the hepcidin gene promoter, subsequently normalizing the reduced expression of FPN. Taken together, the targeting of SMILE by pharmacological agents may provide attractive means to therapeutically control aberrant regulation of iron metabolism responsible for iron overload or iron deficiency.

## Data Availability

Data supporting the findings of this study is contained within the paper and its supplementary material files. Raw data of this study is available from the corresponding author upon reasonable request.

## Supplementary Materials

The following are available online at https://www.mdpi.com/article/10.3390/antiox10101590/s1, Figure S1: Effect of EGCG on viability of hepatocytes, Figure S2: Effect of BMP-6 plus EGCG on viability of hepatocytes, Figure S3: Western blot analysis showing SMAD1/5/8 phosphorylation.

## Abbreviations

EGCG	Epigallocatechin-3-gallate
SMILE	Small heterodimer partner-interacting leucine zipper protein
BMP-6	Bone morphogenetic protein 6
ALK3	Activin receptor-like kinase 3
HJV	Hemojuvelin
SMAD	Small mothers against decapentaplegic homolog protein
FPN	Ferroportin
BMP-RE	BMP-response element
MOI	Multiplicity of infection
IRIDA	Iron-refractory iron deficiency anemia
IL-6	Interleukin-6
ERRγ	Estrogen-related receptor γ
ROS	Reactive oxygen species
FOXO1	Forkhead box protein O1
PCR	Polymerase chain reaction
ELISA	Enzyme-linked immunosorbent assay
Q-PCR	Quantitative PCR
WB	Western blot
HNF4α	Hepatocyte nuclear factor 4α
AMPK	AMP-dependent kinase
LKB1	Liver kinase B1
