# Supplementary material for: Epigallocatechin-3-Gallate Suppresses BMP-6-Mediated SMAD1/5/8 Transactivation of Hepcidin Gene by Inducing SMILE in Hepatocytes
Source: Antioxidants (Basel). 2021 Oct 10;10(10):1590. doi: 10.3390/antiox10101590 (PMC8533173; doi:10.3390/antiox10101590)
Supplement: Supplementary file 1 [file antioxidants-10-01590-s001.zip › antioxidants-1374318-supplementary.pdf]

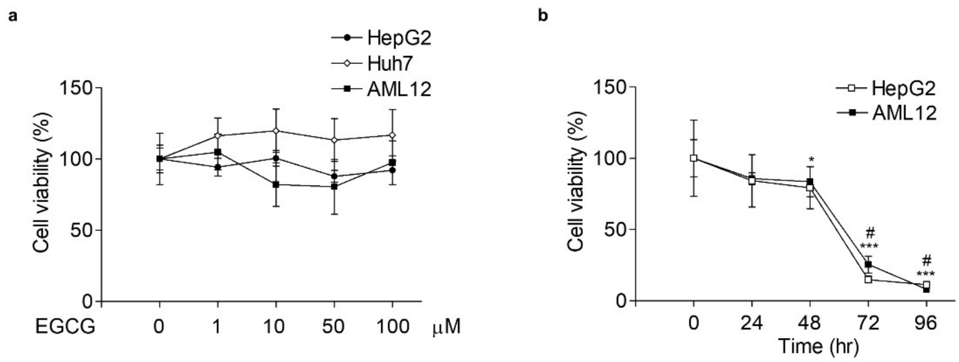

Figure S1. Effect of EGCG on viability of hepatocytes. (a) MTT assay showing effect of EGCG on viabilities of HepG2, Huh7 and AML12 cells in a concentration-dependent manner. HepG2, Huh7, and AML12 cells were treated with various concentrations of EGCG for 12 h. (b) MTT assay showing effect of EGCG on viabilities of HepG2 and AML12 cells in a time-dependent manner. HepG2 and AML12 cells were treated with EGCG (100 μM) up to 96 h. Data are expressed as means ± SD. * compared with at 0 h in HepG2 cell; # compared with at 0 h in AML12 cells. * $P < 0.05$, *** and # $P < 0.001$ by two-tailed Student's *t*-test.

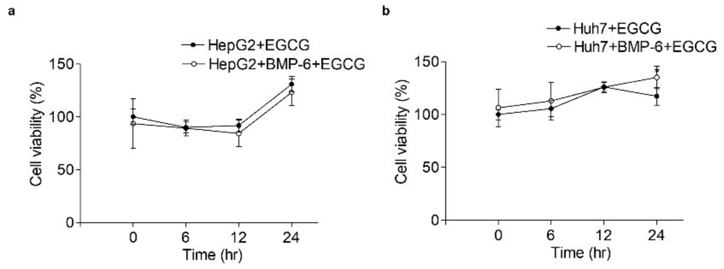

Figure S2. Effect of BMP-6 plus EGCG on viability of hepatocytes. (a-c) MTT assay showing effect of BMP-6 plus EGCG on cell viabilities in a time-dependent manner. HepG2 (a), Huh7 (b) and AML12 (c) cells were treated with EGCG (100 μM) for the indicated time period after pre-treatment with BMP-6 (20 nM) for 12 h.

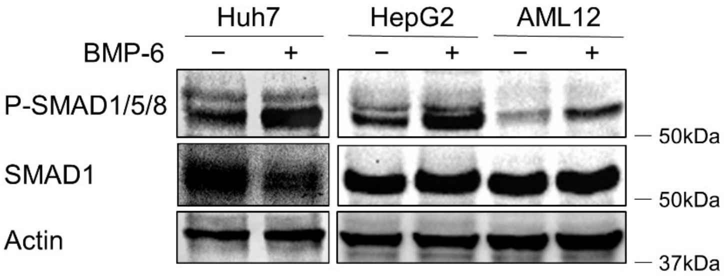

Figure S3. Western blot analysis showing SMAD1/5/8 phosphorylation. Huh7, HepG2 and AML12 cells were treated with recombinant mouse BMP-6 (20 nM) for 12 h.
